# Relationship Between Bruxism and Obstructive Sleep Apnea: A Systematic Review of the Literature

**DOI:** 10.3390/jcm14145013

**Published:** 2025-07-15

**Authors:** Natalia García Doblado, José María Barrera Mora, Francisco Pastor Dorado, Juan C. Rodríguez Fernández, Guillem Ballestero Ordeix, Eduardo Espinar Escalona

**Affiliations:** 1 School of Dentistry, University of Sevilla, 41009 Sevilla, Spain; natgardob@alum.us.es; 2 Orthodontics Section, Department of Stomatology, School of Dentistry, University of Sevilla, 41009 Sevilla, Spain; fpastor@us.es (F.P.D.); jrodriguez21@us.es (J.C.R.F.); eespinar@us.es (E.E.E.); 3 School of Dentistry, University International of Catalunya, 08017 Barcelona, Spain; drgballestero@gmail.com

**Keywords:** sleep bruxism, obstructive sleep apnea, risk factors, polysomnography

## Abstract

**Background and objectives**: The interest in studying the relationship between bruxism and sleep apnea has increased over the past decade, given its prevalence and its implications for both oral and overall health. Bruxism occurs in a significant portion of the population, with an overall incidence ranging between 8 and 31%. Obstructive sleep apnea affects 4–6% of middle-aged men and 2–4% of middle-aged women, and it is associated with diminished quality of life, hypertension, increased cardiovascular risks, traffic accidents, and a higher mortality rate. Although a possible association has been suggested, the causal relationship remains unclear. This review aimed to systematically evaluate the available evidence on the association between SB and OSA, focusing on potential shared risk factors and underlying mechanisms. **Methods:** An electronic literature search was conducted in PubMed, Scopus, Web of Science, and the Cochrane Library for studies published between 2020 and 2025. Inclusion criteria encompassed observational studies and clinical trials involving adults with SB and/or OSA. Risk of bias was assessed using ROBINS-I, and the certainty of evidence was evaluated using GRADE. The review protocol was not registered. **Results**: A total of 11 studies met the inclusion criteria. The prevalence of SB was consistently higher in individuals with OSA compared to the general population. Several studies suggest a potential link through autonomic arousals and neurotransmitter dysregulation. However, inconsistencies in diagnostic criteria and moderate methodological quality limited the strength of the findings. **Conclusions:** There is a notable prevalence of bruxism in patients with OSA, suggesting possible shared pathophysiological mechanisms; however, it is necessary to standardize diagnostic criteria and conduct larger, more standardized studies to clarify the relationship. No funding was received, and the authors declare no conflicts of interest.

## 1. Introduction

Sleep bruxism (SB) and obstructive sleep apnea (OSA) are conditions that have a substantial impact on both oral and general health. The most widely accepted definition of bruxism was formulated in 2013 at an international consensus [1], where it was described as a “repetitive activity of the mandibular muscles characterized by clenching or grinding the teeth and/or bracing or thrusting the mandible.” It can occur while awake (awake bruxism) or during sleep (sleep bruxism) [2]. Both types are regarded as different behaviors, with distinct definitions and multifactorial origins: biological (neurotransmitters, genetics), psychological (stress, anxiety), and exogenous (tobacco, alcohol, drugs).

Recent studies estimate the prevalence of SB to range between 8% and 13% in the general adult population, though self-reported rates may reach higher values when subjective methods are used [1,2,3,4,5,6,7]. Meanwhile, OSA affects approximately 15% to 30% of men and 10% to 15% of women in middle age, depending on the diagnostic criteria used and the population studied [8].

Clinically, bruxism may be harmless, a risk factor, or even a protective factor, depending on the context. For example, it is thought that it may protect the airway in patients with OSA or stimulate salivation in the presence of gastro-esophageal reflux; conversely, it can also give rise to muscular pain, tooth wear, headaches, and damage to oral structures [2,9].

During sleep, SB manifests itself through repetitive rhythmic masticatory muscle contractions (RMMA), which may be phasic, tonic, or mixed [3,9,10,11]. Diagnosis is based on patient interview, clinical examination, intra-oral devices, electromyography (EMG), or polysomnography (PSG), the latter being the gold standard owing to its ability to record multiple physiological parameters during sleep [12].

OSA is characterized by repeated episodes of apnea and hypopnea that are caused by collapse of the upper airway, leading to hypoxemia and hypercapnia accompanied by micro-arousals and alterations in sleep architecture [13,14]. OSA is diagnosed when the apnea–hypopnea index (AHI) is ≥15 events·h^−1^, or ≥5 events·h^−1^ if there are symptoms such as excessive daytime sleepiness or fatigue [15].

The pathogenesis of OSA is highly heterogeneous and is influenced by anatomical factors, impairment of the pharyngeal dilator muscles, ventilatory control instability, and a low arousal threshold. Lung volume, fluid redistribution, and arousal intensity also play a role [15,16,17,18,19,20,21,22]. The main risk factors include male sex, middle age, obesity, alcohol, tobacco, and sleeping in the supine position [13].

The typical OSA cycle comprises apnea–hypopnea, blood-gas alterations, micro-arousal, and restoration of airflow, which fragments sleep and causes daytime somnolence, snoring, fatigue, headaches, and other symptoms. In severe cases, patients may experience road-traffic accidents and cardiovascular, neuro-cognitive, and metabolic consequences [13].

The terminology has evolved over time. At present the preferred term is “obstructive sleep apnea” (OSA), omitting words such as “syndrome” or “hypopnea” to better reflect the nature of the disorder [14].

Despite prior efforts to clarify the link between sleep bruxism and obstructive sleep apnea, current evidence remains inconclusive. For instance, the scoping review by Pauletto et al. [16] found no consistent association in adult populations, largely due to variability in study designs and diagnostic approaches. Likewise, the systematic review and meta-analysis conducted by Błaszczyk et al. [17] did not find a statistically significant relationship between the two conditions, noting, however, that the methodological quality of the included studies was generally low. These findings highlight the ongoing need for more current and methodologically robust investigations.

The aim of the present study was to evaluate the available scientific evidence on the relationship between sleep bruxism (SB) and obstructive sleep apnea (OSA) through a systematic literature review. Three specific objectives were set: (1) to analyze the pathophysiological mechanisms that could explain the relationship between SB and OSA, assessing whether there is a causal link or mere coexistence; (2) to examine the role of risk factors in the development of bruxism in patients with OSA; and (3) to assess the methodological quality of the studies included.

Lastly, based on the analysis of the selected papers, a conclusion will be drawn that synthesizes the most relevant findings.

## 2. Materials and Methods

### 2.1. Protocol and Registration

This systematic review adheres to the criteria laid down in the PRISMA 2020 (Preferred Reporting Items for Systematic Reviews and Meta-Analyses) statement.

The review protocol was not submitted to a public registry owing to time limitations.

### 2.2. P.I.C.O. Question

The cornerstone of the review was the formulation of a specific, structured clinical question using the key concepts that would guide and facilitate the search for, and retrieval of, the most notable and pertinent information required to carry out this systematic review (Table 1).

### 2.3. Inclusion and Exclusion Criteria

To enable an appropriate selection of articles retrieved in the various searches, the following inclusion and exclusion criteria were established.

#### 2.3.1. Inclusion Criteria

This review included original research articles that met the following conditions: (A) studies conducted in human populations, including prospective or retrospective designs, randomized clinical trials, observational studies, and case–control studies; (B) only articles published between 2020 and 2025 were included to ensure the relevance of the data and manage the high volume of publications available on the topic, thus avoiding excessive heterogeneity in study design and diagnostic criteria; (C) studies involving patients diagnosed with bruxism, obstructive sleep apnea, or both; (D) publications explicitly addressing the relationship between bruxism and sleep apnea; and (E) studies providing relevant data or findings that contribute to the objectives of this review.

#### 2.3.2. Exclusion Criteria

We excluded studies based on the following criteria: (A) research conducted in non-human models or pediatric populations; (B) publication types such as letters, author responses, editorials, case reports, case series, pilot studies, meta-analyses, and other systematic reviews; (C) duplicate articles identified across databases to avoid redundant data; (D) studies with poorly described methodologies or insufficient detail for evaluation; and (E) articles whose content was not directly related to the focus of this systematic review.

No language restrictions were imposed.

### 2.4. Information Sources and Search Strategy

To identify the studies included in our work, several bibliographical searches were performed in the main scientific databases: PubMed, Scopus, the Cochrane Library, and Web of Science.

The following search strategies were designed, using controlled vocabulary terms aligned with the topic of the review and combined strategically with Boolean operators:

The terms “Bruxism” OR “Sleep Bruxism” were combined with “Risk Factors” OR “Occlusal risk factors” to identify studies focusing on possible contributing elements.

A broader search linked “Bruxism” OR “Sleep Bruxism” with “Sleep Apnea Syndromes” OR “Obstructive Sleep Apnea” and “Risk Factors” OR “Occlusal risk factors”, to explore shared or overlapping risk profiles.

To capture studies addressing coexisting conditions, the terms “Bruxism” OR “Sleep Bruxism” were paired with “Obstructive Sleep Apnea” and “Comorbidity.”

Additionally, searches combined “Bruxism” OR “Sleep Bruxism” with the term “Comorbidity” to detect broader associations.

Finally, we used the combination “Bruxism” OR “Sleep Bruxism” with “Obstructive Sleep Apnea” and “Relationship” to identify studies directly analyzing the interaction between the two conditions.

All retrieved results were exported to Zotero for management, and duplicates were systematically removed prior to screening.

The full methodological details can be found in Supplementary Table S1, included in the Supplementary Material File. 

### 2.5. Study Selection and Data Extraction Process

The initial screening of titles and abstracts was performed independently by two reviewers, who applied the predefined inclusion and exclusion criteria. Disagreements during this phase were discussed until a consensus was reached. Subsequently, the full texts of potentially eligible articles were assessed independently by the same reviewers to confirm their relevance. Any discrepancies were resolved through joint review and consensus.

### 2.6. Data Charting and Level of Evidence

For each study included in this systematic review, the following data were collected: title and author, journal and year of publication, study design and level of evidence, purpose of the research, materials and methods used, as well as sample size, mean age, and main conclusions.

To determine the level of evidence of the analyzed articles, we used the classification established by the Oxford Centre for Evidence-Based Medicine (OCEBM). The GRADE system was also used to assess the certainty of the evidence.

### 2.7. Risk of Bias in Individual Studies

To assess the quality of the information contained in the studies, we employed the ROBINS-I (Risk of Bias in Non-Randomized Studies of Interventions) tool, designed to evaluate the risk of bias in observational studies (see Supplementary Table S2 for detailed assessment.).

### 2.8. Risk of Bias Across the Included Studies—Certainty of Evidence Assessment

The GRADE (Grading of Recommendations, Assessment, Development, and Evaluations) tool was applied to assess the associated certainty of evidence.

## 3. Results

### 3.1. Study Selection

**Figure 1:**
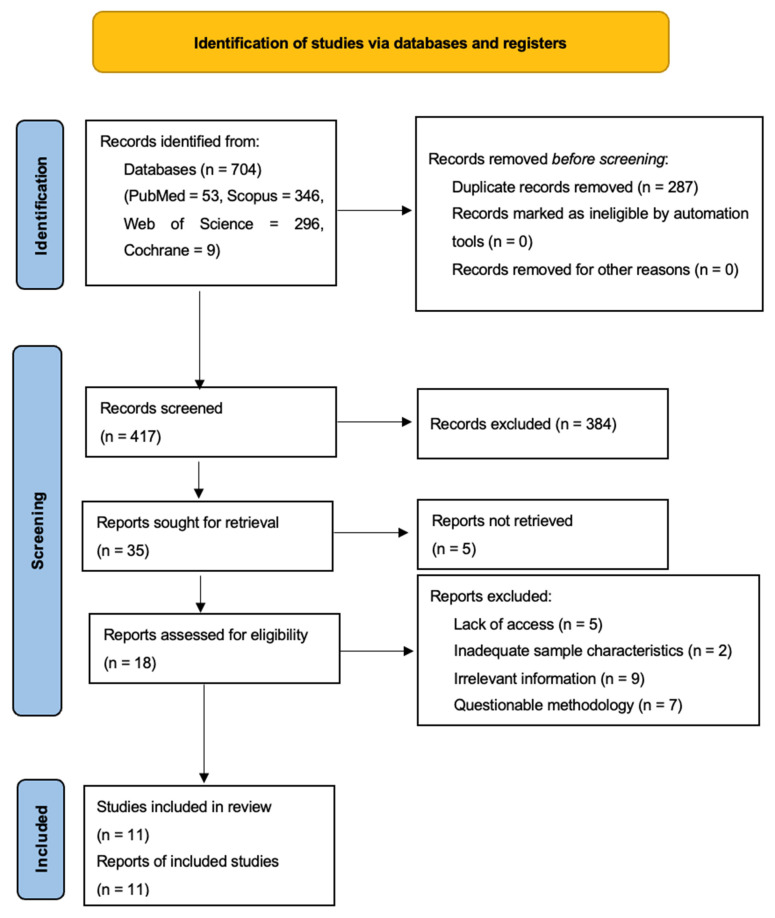
Final flow diagram.

Figure 1 shows the PRISMA flow diagram, which clearly, structurally, and concisely depicts the study-selection process.

The initial search of several databases yielded 704 results, which were exported to Zotero reference manager (version 7.0.15, 64-bit). After removing 287 duplicate records, 417 articles remained. Of these, 337 were excluded after title screening and 47 after reviewing the abstract, as they did not meet the inclusion–exclusion criteria or provided information that was not relevant to the review. At this stage, 35 full-text articles remained for eligibility assessment.

Finally, 5 articles were excluded owing to lack of access, 7 because of questionable methodology, 2 because of sample characteristics and 9 for not containing significant information, resulting in a total of 11 articles included in the review. Only one reviewer conducted the study selection process.

### 3.2. Study Characteristics

Of the eleven articles included, one [23] was a prospective clinical trial and ten were observational studies [24,25,26,27,28,29,30,31,32,33]. Of these latter studies, five are cross-sectional [26,27,28,30,31], three are case–control [25,32,33], and two are cohort studies [24,29]. All are written in English.

Eight of the studies analyze the relationship between bruxism and sleep apnea [26,27,28,29,30,31,32,33], and some of them even explore these conditions together with alcohol consumption, antidepressant therapy, or insomnia syndrome. Two others compare the effect of treatment with MAD [23] and PAP [24] patients with bruxism and apnea, and the remaining article assesses sleep-architecture conditions in patients with bruxism [25].

Table 2 summarizes the relevant information from each of the included studies, detailing the title, authors, journal and year of publication, study type, level of evidence, objective, materials and methods used, sample size, and mean age, as well as the main conclusions.

### 3.3. Risk of Bias in the Articles and Level of Evidence

The different domains used to evaluate bias for each of the studies are presented in Supplementary Table S2 for a detailed assessment. We applied the ROBINS-I assessment scale. All our articles have a “moderate” risk of bias.

Among the observational studies, the most common source of bias is outcome measurement, either because a single investigator is responsible for the evaluations or because it is not specified. We also found participant selection bias, where specific clinical samples or small samples are chosen, as well as measurement bias due to the lack of standardization in defining and quantifying phenomena such as masticatory activity (rhythmic masticatory muscle activity [RMMA] or jaw closing muscle activity [JCMA]) and sleep apnea.

The Oxford Centre for Evidence Based Medicine (OCEBM) classification is a tool that allows us to grade the level of evidence according to each clinical setting. This systematic review carries a “moderate level of evidence” regarding the association between sleep bruxism and obstructive sleep apnea.

This review did not include a specific assessment of reporting bias, such as potential publication bias or selective outcome reporting. Given the absence of a meta-analytical synthesis and the lack of registered protocols in the included studies, standard approaches like funnel plot analysis were not applicable. We recognize this as a methodological limitation that could influence the completeness of the evidence.

### 3.4. Risk of Bias Across the Included Studies—Certainty of Evidence Assessment

The GRADE (Grading of Recommendations, Assessment, Development and Evaluation) methodology was applied to assess the certainty of the evidence for the main findings. The certainty of the evidence was classified as low for the association between bruxism and obstructive sleep apnea (OSA), due to the inconsistency of results across studies and the variability in diagnostic criteria. As no meta-analyses were performed in this review, we did not apply formal statistical methods to detect potential bias due to missing results for each synthesis. Nevertheless, we acknowledge that some results may be underreported due to publication bias or selective reporting, particularly in studies without protocol registration or prespecified outcomes.

## 4. Discussion

### 4.1. Method

The selected articles show heterogeneity in methodology and diagnostic criteria. In general, all the studies use overnight polysomnography (most frequently for a single night, except Aarab et al. [23] which records two nights) as the “gold standard” for diagnosing both apnea and bruxism. Several employ additional tools such as audio and video recordings (Smardz et al., Dadphan et al., Okura et al., Holanda et al., Cid-Verdejo, and Wieckiewicz et al. [24,25,30,31,32,33]) and self-report questionnaires in Maluly et al. [27], Holanda et al. [25], Cid-Verdejo et al. [33] (Epworth Sleepiness Scale, Pittsburgh Sleep Quality Index, Insomnia Severity Index, etc.) and Ning et al. [29] (which also used CBCT, intra-oral images, clinical examination, and visual analogue scales for subjective pain assessment), and even real-time PCR amplification with melting-curve analysis in Wieckiewicz et al. [32].

At the sample level, Maluly et al. [27] (EPISONO, *n* = 1042), stands out; in addition to having a large sample, the study is based on a general-population cohort, whereas the others analyze specific clinical samples.

A threshold of “≥2 episodes/hour” or “≥2 RMMA/hour” is usually used to classify the “presence of SB”. Some articles (Dadphan et al., Li et al., Cid-Verdejo et al., Smardz et al., Wieckiewicz et al. [24,26,31,32,33]) also set a threshold of “≥4 episodes/hour” to classify severe bruxism. Others distinguish “possible” bruxism (self-reported only) from “definite” bruxism (confirmed by PSG) (Maluly et al. [27]).

The studies included in this review exhibit considerable heterogeneity in their diagnostic approaches to both OSA and SB. While most authors adhere to the diagnostic criteria established by the American Academy of Sleep Medicine (AASM) for OSA—specifically, an apnea–hypopnea index (AHI) ≥5 with symptoms or ≥15 regardless of symptoms—there is notable variability in how SB is defined and quantified. Some studies rely solely on polysomnography (PSG), the gold standard, while others include self-report questionnaires or combine EMG with audio-video recordings. This inconsistency complicates the comparison of results and weakens the strength of the overall evidence.

Although earlier reviews have examined the possible link between sleep bruxism and obstructive sleep apnea, their conclusions remain limited by methodological concerns. The scoping review by Pauletto et al. [16] highlighted the lack of standardized diagnostic criteria and the broad variability in study designs as key barriers to reaching firm conclusions. Similarly, Błaszczyk et al. [17], in a recent meta-analysis, found no significant association between the two conditions but acknowledged that the overall quality of the available studies was low and the diagnostic approaches inconsistent. These limitations emphasize the need for more targeted analyses, such as the present review, which focuses on recent studies and evaluates them using consistent inclusion criteria and structured methodological appraisal.

### 4.2. Prevalence and Risk Factors

#### 4.2.1. Prevalence of Bruxism in Patients with OSA

A high prevalence of sleep bruxism (SB) in patients with obstructive sleep apnea (OSA) is a repeated finding in several studies. Li et al. [26] found SB in 49.7% of adults with OSA, far above the general prevalence (8–13%). Similarly, Dadphan et al. [24] recorded about 49% SB, although split-night polysomnography may have influenced their results. Maluly et al. [27] showed that in the general population the coexistence of SB with OSA (9–12.3%) and insomnia symptoms (6.4%) is high, suggesting an influence of sex, age, and sleep quality. Massahud [28] diagnosed SB in 42.9% of 240 patients undergoing full polysomnography, whereas Okura et al. [30] reported 28% in a smaller sample.

#### 4.2.2. Demographic and Clinical Risk Factors

Several studies associate certain factors with a higher risk of bruxism (SB) in patients with OSA. Li et al. [26] found that being male and having a relatively low BMI increase the likelihood of SB, whereas Holanda et al. [25] indicate that obese individuals are less often diagnosed with SB. Dadphan et al. [24] underscore the relevance of self-perceived tooth grinding to the presence of bruxism

Alcohol consumption, insomnia, and antidepressant therapy are thought to be related to an increased risk or episodes of SB in OSA patients. Regarding alcohol, it affects sleep consolidation and alters sleep architecture, leading to a sudden increase in serotonin and dopamine that may modulate orofacial muscle activity. Maluly et al. [27] emphasize that insomnia, especially in middle-aged women, is significantly associated with SB. Significant risk factors for “definite” SB were insomnia syndrome and overweight. Massahud et al. [28] suggest that SB could be a side-effect of antidepressants such as Selective Serotonin Reuptake Inhibitors (SSRIs) and Serotonin-Norepinephrine Reuptake Inhibitor (SNRIs), possibly through alterations in dopaminergic activity modulated by neurotransmitters like serotonin, noradrenaline, and histamine. However, they found no significant differences regarding antidepressant use between individuals with and without bruxism.

#### 4.2.3. Influence of Sleep Position and OSA Severity

Smardz et al. [31] found that the supine-position OSA phenotype is associated with higher incidence and severity of bruxism (SB), although adjusting for age and BMI removed the independent relationship. Conversely, Dadphan et al. [24] identified that a higher respiratory disturbance index (RDI) in the non-supine position increases the risk of SB.

Maluly et al. [27] determined that being male, overweight/obese, having an AHI above 30, and insomnia are risk factors for SB; overweight and insomnia showed significant associations. Massahud et al. [28] reported SB more frequently in men and in those with severe OSA, with these patients exhibiting higher mean AHI values. Ning et al. [29] suggested that as OSA worsens, dental wear, orofacial pain, and SB increase, and observed a negative correlation between AHI and condylar volume/area—that is, as AHI values rose, condylar volume decreased.

In contrast, Holanda et al. [25] and Cid-Verdejo [31] reported that OSA presence decreases the odds of SB, as patients without bruxism showed significantly higher AHI and RDI values, possibly due to more arousals triggered by respiratory events [25]. In the study by Cid-Verdejo [33], bruxism episodes were more frequent in patients without OSA or with mild OSA.

### 4.3. Pathophysiological Mechanisms

#### 4.3.1. Compensatory Response and Protective Function

One of the most discussed theories on the relationship between SB and OSA is that the former may act as a compensatory or defensive mechanism against episodes of airway obstruction. Ning et al. [29] and Cid-Verdejo [33] propose that SB may have a protective role by promoting mandibular protrusion and re-establishing upper-airway patency. This would be explained by activation of mandibular-depressor muscles (e.g., genioglossus), which generate rhythmic movements that trigger RMMA, opening the airway. Thus, SB could be a secondary manifestation of OSA. However, authors cited by Ning et al. [29] such as Sjöholm, reported that this mechanism is ineffective in severe OSA, where diminished sleep depth limits the protective response.

#### 4.3.2. Relationship Between Arousals, Respiratory Events and RMMA

The relationship between micro-arousals, sleep bruxism (SB), and apneic events has gained increasing attention in recent literature as a possible key to understanding the interaction between SB and obstructive sleep apnea (OSA). Research by authors such as Okura et al. [30] and Li et al. [26] suggests a temporal closeness between respiratory events and the initiation of rhythmic masticatory muscle activity (RMMA). These observations lend support to the theory that brief cortical arousals—often induced by episodes of oxygen desaturation or carbon dioxide buildup—may create a favorable context for the emergence of bruxism episodes. Although RMMA does not always correspond directly to respiratory disturbances, an increased arousal threshold combined with elevated sympathetic nervous system activity may contribute to its expression. Within this framework, SB could be interpreted as a physiological response to sleep disruption, potentially playing a protective or adaptive role in less severe cases of OSA.

Aarab et al. [23] showed that mandibular advancement devices (MAD) significantly reduce masticatory muscle activity associated with respiratory arousals, suggesting that SB could be a compensatory response to re-establish the airway and prevent hypoxia. This response occurs after a cortical micro-arousal caused by hypoxia and hypercapnia during obstructive events [23,24,29]. According to Smardz [31], this “arousal” involves an abrupt change in brain activity without full consciousness, accompanied by increased sympathetic tone and muscle contraction, including mandibular muscles. Under this hypothesis, SB is secondary to the arousal. By improving airway patency, MAD could reduce both obstructive events and RMMA, though the theory remains debated.

Okura et al. [30] found that RMMA within 10 s after respiratory events was more frequent in patients with OSA and SB than in those without SB, but without correlation with the AHI, unlike non-specific masticatory muscle activity (NSMA), which did correlate with AHI. This suggests a distinct physiological phenotype in patients with OSA and associated SB.

Li et al. [26] observed that although RMMA did not directly correlate with AHI or micro-arousals, 85.7% of SB episodes occurred within 5 s after an arousal. Moreover, RMMA was more often linked to non-respiratory than respiratory arousals, with similar proportions in people with and without OSA in other studies. This suggests that SB is not directly due to obstruction but is an autonomic response linked to arousal. Thus, arousal acts as a “permissive window” for RMMA, not as a direct trigger.

#### 4.3.3. Neurophysiological Factors, Neurotransmitters and Genetics

According to Massaud et al. [28], fluctuations in serotonin, dopamine, and opioid release can induce rhythmic movements of the masticatory muscles, facilitating mandibular protrusion and airway opening. Thus, SB could be viewed as a positive response at the end of obstructive events, helping to restore ventilation.

Wieckiewicz et al. [32] analyzed single-nucleotide polymorphisms (SNPs) of genes such as HTR2A, COMT, and DRD1, which regulate dopaminergic and serotonergic pathways, in patients with OSA. The SNP rs6313 of HTR2A (TT homozygotes) was associated with a higher bruxism index, whereas COMT rs4680 and DRD1 rs686 showed no significant associations. They did find a correlation between the bruxism episode index (BEI) and AHI in patients carrying the T allele of SNP rs2770304 (HTR2A), suggesting a genetic predisposition to masticatory activation in response to arousals.

### 4.4. Sleep Architecture and OSA Phenotypes

#### 4.4.1. Impact on Sleep Stages

SB can influence sleep structure. Studies by Holanda et al. [25] and Okura et al. [30] found significant differences between patients with and without SB. Bruxers showed shorter wake time after sleep onset (WASO), shorter N1 duration, longer N3 (deep sleep) duration, and a higher proportion of REM sleep.

Holanda et al. [25] reported that individuals with SB had less fragmented sleep, fewer arousals, and significantly lower AHI and RDI values. These findings suggest that bruxers have deeper, more restorative sleep, possibly as a compensatory mechanism for mandibular activity (RMMA). Li et al. [26] noted a higher proportion of NREM 1 sleep in SB-OSA patients, though not statistically significant. NREM 3 involves key processes of cellular repair and immune strengthening, which could explain its longer duration in bruxers.

Okura et al. [30] found a higher percentage of REM sleep in patients with OSA and SB, and a reduction of AHI in REM among SB patients. They observed increased SB in NREM sleep, without significant correlation.

#### 4.4.2. OSA Phenotypes and Sleep Bruxism

Smardz et al. [31] analyzed SB incidence across OSA phenotypes, concluding that position-dependent OSA shows higher SB prevalence and severity, possibly due to anatomical reduction of airflow in the supine position. The REM-dependent phenotype showed no significant relationship with SB, and adjusted analysis revealed no clear independent association between OSA and SB. REM-related OSA was linked to generally lower AHI scores and reduced severe OSA incidence; overall, SB is reported more frequently during NREM stages. Therefore REM-related OSA is less likely to coincide with bruxism. Although theoretically SB should act as a response in both phenotypes, significant association was confirmed only for position-dependent OSA, perhaps because REM sleep represents a relatively small proportion of the sleep cycle.

Dadphan et al. [24] warned that a high RDI in non-supine positions can also be a risk factor for SB associated with OSA, indicating that these positions do not fully eliminate the risk of obstructive events or bruxism.

### 4.5. Therapeutic Interventions for OSA and Their Effects on SB

#### 4.5.1. Mandibular Advancement Devices (MAD)

Currently, MADs are indicated for mild or moderate OSA and for severe OSA when CPAP is not tolerated. Aarab et al. [23] demonstrated that MAD significantly reduces masticatory activity associated with respiratory arousals. Its efficacy lies in mandibular and lingual advancement, increasing airway patency by widening it and preventing collapse during sleep.

With MAD in situ, a significant decrease was observed in masticatory activity linked to arousals. However, the total masticatory activity index (JCMA = RMMA + OFA) did not change, probably because respiratory arousals account for roughly 50% of the total.

These results support that MAD therapy reduces JCMA associated with respiratory arousals, suggesting that these muscle activations are more related to arousals than to the respiratory events themselves.

#### 4.5.2. Positive Airway Pressure (PAP)

Positive airway pressure (PAP) is another effective therapy for OSA. Dadphan et al. [24] showed that PAP significantly reduces the SB episode index (BEI). With optimal PAP, only 20% of patients maintained significant SB (BEI ≥ 2). About 50% of OSA patients exhibited SB, and 73.5% of episodes were associated with respiratory events occurring within 5 s.

These findings support that SB could be secondary to respiratory instability and the arousals it generates. No differences were observed in the arousal index between the groups with and without SB, although using split-night polysomnography might have influenced the results.

### 4.6. Limitations

This systematic review has limitations that should be considered when interpreting the results. The lack of homogeneity in the diagnostic criteria for SB and OSA makes comparison between studies difficult and weakens the conclusions. Research with larger samples, longer duration (more nights of PSG) and prospective designs is needed to establish causal relationships between SB and OSA. Furthermore, all the included studies present some degree of bias, mainly in variable control, outcome measurement, and participant selection, which can affect their internal validity.

## 5. Conclusions

In summary, the evidence reviewed suggests that there is evidence of a high prevalence of sleep bruxism (SB) in patients with obstructive sleep apnea (OSA), exceeding the estimates for the general population. Although the exact nature of this relationship remains uncertain, several studies point to shared pathophysiological mechanisms, including autonomic response, cortical arousal, and possible genetic bases in dopaminergic and serotonergic pathways are the main hypotheses on the SB-OSA relationship, but more evidence is required.

Various risk factors (male sex, alcohol consumption, insomnia, antidepressant use, and overweight/obesity) are associated with increased SB episodes in the context of OSA, though not all studies agree. Body position during sleep can influence the frequency and intensity of both OSA and SB, but results differ some indicate higher risk in the supine position, others note greater impact in non-supine positions. Moreover, variations in sleep architecture are observed: some bruxers have more deep sleep (N3) and fewer arousals, while other studies find no statistically significant associations or observe differences according to REM or NREM phases. Therapeutic interventions for OSA, such as mandibular advancement devices (MAD) or positive airway pressure (PAP), can reduce bruxism activity.

Despite these findings, inconsistencies in diagnostic criteria and methodological heterogeneity among the included studies limit the strength of the conclusions. There is a clear need for prospective research using standardized definitions and multi-night PSG to clarify causality and improve clinical management of patients presenting with both conditions.

## Figures and Tables

**Table 1:** P.I.C.O. question.

P (Patient)	I (Intervention)	C (Comparison)	O (Outcome)
“Sleep Bruxism” AND “Sleep Apnea Syndromes”	“Observation relationship”	“Control Groups”	“Risk factors”

**Table 2:** Summary of the selected studies.

Title/Author	Journal/Year	Study Type	Level of Evidence	Objective	Materials and Methods	Conclusions
The effects of mandibular advancement appliance therapy on jaw-closing muscle activity during sleep in patients with obstructive sleep apnea: a 3–6 months follow-up (Aarab et al.) [23]	Journal of Clinical Sleep Medicine, (2020)	Randomized controlled trial	1b	To investigate the effects of MAD therapy on jaw-closing muscle activity (JCMA) related to respiratory arousals in OSA.	18 patients (5 women, 13 men) with OSA; two PSG recordings (with and without MAD). Mean age 49.4 ± 9.8 years.	MAD therapy significantly reduced mandibular muscle activity associated with respiratory arousals in patients with OSA.
Prevalence and predictors of sleep bruxism in patients with obstructive sleep apnea and the effect of positive airway pressure treatment (Dadphan et al.) [24]	Sleep and Breathing (2024)	Cohort Study	3b	To determine the prevalence and risk factors of SB in patients with OSA and to compare SB episodes before and during positive airway pressure (PAP) therapy.	Retrospective review of 100 OSA patients (73 men, 27 women) who underwent split-night PSG and optimal PAP. SB group: 49 patients (33 men and 16 women) Non-SB group: 51 patients (40 men and 11 women) Mean age: 50.8 ± 16.7 years	About 49% of patients had SB, mostly associated with OSA; optimal PAP significantly reduced SB episodes.
Sleep architecture and factors associated with sleep bruxism diagnosis scored by polysomnography recordings: A case-control study (Holanda et al.) [25]	Archives of Oral Biology, (2020)	Case–control Study	3b	To assess the association between sleep architecture, clinical conditions and SB diagnosis using PSG recordings.	PSG records of 58 bruxers and 58 controls from a private clinic. Cases: 33 women, 25 men; mean age 42.2 ± 15.5 years. Controls: 33 women, 25 men; mean age 42.6 ± 14.8 years	SB diagnosis was significantly associated with BMI, alcohol consumption, and changes in sleep parameters (WASO, N1, N3, etc.).
“Sleep bruxism is highly prevalent in adults with obstructive sleep apnea: a large-scale polysomnographic study” (Li et al.) [26]	Journal of Clinical Sleep Medicine (2023)	Cross-sectional study	3b	To determine the prevalence and risk factors of SB in adults with OSA and to analyze the relationship between SB, arousals and respiratory events.	Overnight full PSG in 914 adults (305 women, 609 men) with OSA. SB group: 454 patients (126 women and 328 men) Non-SB group: 460 patients (179 women and 281 men) Mean age: 53 ± 17 years	49.7 % of adults with OSA had SB. Male sex, lower BMI, and a higher percentage of N1 sleep were associated with a greater SB risk.
“Sleep bruxism and its associations with insomnia and OSA in the general population of Sao Paulo” (Maluly et al.) [27]	Sleep Medicine (2020)	Cross-sectional study	3b	To evaluate the association between SB, insomnia, and OSA in the general population.	Data from the EPISONO study (*n* = 1042; 575 men, 467 women) based on questionnaires and PSG. Group A “Possible SB”: 127 patients (47 men and 80 women) out of 1042 Group B “Definite SB”: 56 patients (22 men and 34 women) out of 620 Mean age: Between 20–80 years	Insomnia is likely associated with SB, especially in middle-aged women, whereas the relationship with OSA varies by age and sex.
“Association between sleep bruxism, use of antidepressants, and obstructive sleep apnea syndrome: A cross-sectional study” (Massahud et al.) [28]	Journal Oral Rehabilitation (2022)	Cross-sectional study	3b	To investigate the association between SB, antidepressant use and obstructive sleep apnea–hypopnea syndrome (OSAHS).	240 patients (118 men, 122 women) underwent full PSG and clinical data collection on antidepressant use. SB group: 103 patients (62 men and 41 women) Non-SB group: 137 patients (60 men and 77 women) Mean age: 51.75 ± 15.77 years	The relationship between antidepressant use and SB was inconclusive. SB was mainly associated with severe OSAHS.
“Obstructive sleep apnea: a follow-up program in its relation to temporomandibular joint disorder, sleep bruxism and orofacial pain” (Ning et al.) [29]	BMC Oral Health (2023)	Cohort study	3b	To evaluate the relationship between OSA, TMD, SB, and orofacial pain over time.	Follow-up of 71 OSA patients (45 men, 26 women) assessed at three time-points with PSG, clinical exams, and CBCT. Mean age: 36 ± 3.5 years	Moderate-to-severe OSA worsens orofacial pain and dental wear, affects TMJ volume/surface area, and can change condylar position; proper OSA therapy may alleviate these effects.
“Relationships between respiratory and oromotor events differ between motor phenotypes in patients with obstructive sleep apnea” (Okura et al.) [30]	Frontiers in Neurology (2023)	Cross-sectional study	3b	To investigate the relationship between SB and OSA in relation to sleep architecture.	PSG of 36 OSA patients; comparison of sleep, respiratory and oromotor variables between those with and without SB. OSA group: 26 patients (20 men and 6 women). Mean age: 49.7 ± 14.1 years. OSA+SB group: 10 patients (9 men and 1 woman). Mean age: 49.3 ± 15.2 years.	OSA patients with SB show a unique phenotype with a higher REM percentage, lower REM AHI, and greater RMMA linked to respiratory events.
Is there an association between sleep bruxism and obstructive sleep apnea? A case-control polysomnographic investigation. (Cid-Verdejo et al.) [33]	Sleep Medicine (2024)	Case–control study	3b	To estimate the association between sleep bruxism (SB) and obstructive sleep apnea (OSA) based on the severity of the latter.	37 patients (24 men and 13 women) with and without OSA underwent full-night PSG. Case group: 37 patients (16 without OSA and 21 without SB) Control group: 37 patients (21 with OSA and 16 with SB) Mean age: 49.63 ± 11.59 years	In patients with subclinical and mild OSA, SB might play a certain protective role.
“Incidence of Sleep Bruxism in Different Phenotypes of Obstructive Sleep Apnea” (Smardz et al.) [31]	Journal of Clinical Medicine (2022)	Cross-sectional study	3b	To assess the incidence of SB in different OSA phenotypes.	Video PSG of 179 patients: 94 position-dependent, 85 REM-related. Position-related OSA group: 34 women and 60 men. Mean age: 52.19 ± 13.39 years REM-related OSA group: 28 women and 57 men. Mean age: 51.95 ± 13.39 years	Position-dependent OSA seems associated with a higher incidence of SB and severe SB, but the relationship is not independent.
“Genetic basis of sleep bruxism and sleep apnea—response to a medical puzzle” (Wieckiewicz et al.) [32]	Scientific Reports (2020)	Case–control study	3b	To evaluate the association of specific single-nucleotide polymorphisms (SNPs) in serotonin- and dopamine-pathway genes with SB and OSA and to explore their relationship.	PCR-based genetic analysis of 100 patients (with SB and/or OSA) and 125 controls testing SNPs rs2770304 and rs6313 (HTR2A), rs4680 (COMT) and rs686 (DRD1). Case group: 69 men and 31 women. Mean age: 35.2 ± 11.41 years Control group: 62 men and 63 women. Mean age: 29.98 ± 9.23 years	Possible genetic contribution of the serotonin receptor gene HTR2A to SB etiology; DRD1 rs686 may potentially influence SB risk.

Abbreviations: W: woman; M: man; BMI: body mass index; MAD: mandibular advancement device; PAP: positive airway pressure; AHI: apnea–hypopnea index; SB: sleep bruxism; OSA: obstructive sleep apnea; OSAHS: obstructive sleep apnea-hypopnea-syndrome; JCMA: jaw-closing muscle activity; RMMA: rhythmic masticatory muscle activity; SNP: single-nucleotide polymorphisms; PSG: polysomnography; WASO: wake time after sleep onset.

## Data Availability

The data are public. All the information contained in the article is supported by the bibliographic references, which are available in the databases used for the systematic review.

## Supplementary Materials

The following supporting information can be downloaded at: https://www.mdpi.com/article/10.3390/jcm14145013/s1, Table S1: Results according to database and search strategy used. Table S2: Risk of bias according to the ROBINS-I quality assessment scale.
