# Supplementary material for: Relationship Between Bruxism and Obstructive Sleep Apnea: A Systematic Review of the Literature
Source: J Clin Med. 2025 Jul 15;14(14):5013. doi: 10.3390/jcm14145013 (PMC12295005; doi:10.3390/jcm14145013)
Supplement: Supplementary file 1 [file jcm-14-05013-s001.zip › jcm-3766448-supplementary.pdf]

Journal of
Clinical Medicine

MDPI

Systematic Review

Relationship Between Bruxism and Obstructive Sleep Apnea: A Systematic Review of the Literature

Natalia García Doblado, José María Barrera Mora, Francisco Pastor Dorado, Juan C. Rodríguez Fernández, Guillem Ballestero Ordeix and Eduardo Espinar Escalona

This supplementary file includes additional tables referenced in the main manuscript. These provide detailed descriptions of the search strategy (Supplementary Table S1) and the risk of bias according to the ROBINS-I quality assessment scale (Supplementary Table S2).

Supplementary Table S1. Results according to database and search strategy used.

SEARCH STRATEGY	PUBMED	SCOPUS	COCHRANE	WEB OF SCIENCE
("Bruxism" OR "Sleep Bruxism") AND ("Risk Factors" OR "Occlusal risk factors")	30	177	5	211
("Bruxism" OR "Sleep Bruxism") AND ("Sleep Apnea Syndromes" OR "Obstructive sleep apnea") AND ("Risk Factors" OR "Occlusal risk factors")	4	20	1	18
("Bruxism" OR "Sleep Bruxism") AND ("Sleep Apnea Syndromes" OR "Obstructive sleep apnea") AND "Comorbidity"	4	25	1	8
("Bruxism" OR "Sleep Bruxism") AND "Comorbidity"	9	86	1	24
("Bruxism" OR "Sleep Bruxism") AND ("Sleep Apnea Syndromes" OR "Obstructive sleep apnea") AND "Relationship"	6	38	1	35
SUBTOTAL	53	346	9	296
TOTAL	704			

https://doi.org/10.3390/jcm14145013

Supplementary Table S2. *Risk of bias according to the ROBINS-I quality assessment scale.*

AUTHORS / YEAR		FIELDS							OVERALL RISK
		**PRE-INTERVENTION**		**INTERVENTION**	**POST-INTERVENTION**				
		BIAS DUE TO CONFOUNDING	BIAS IN PARTICIPANT SELECTION	BIAS IN CLASSIFICATION OF INTERVENTIONS	BIAS DUE TO DEVIATIONS FROM INTENDED INTERVENTIONS	BIAS DUE TO MISSING DATA	BIAS IN OUTCOME MEASUREMENT	BIAS IN SELECTION OF THE REPORTED RESULT	
Aarab et al.	**2020**	(1)	(2)	(1)	(1)	(2)	(2)	(1)	(2)
Dadphan et al.	**2024**	(2)	(2)	(1)	(1)	(1)	(2)	(1)	(2)
Holanda et al.	**2020**	(2)	(2)	(1)	(1)	(2)	(1)	(1)	(2)
Li et al.	**2023**	(2)	(2)	(1)	(1)	(1)	(1)	(1)	(2)
Maluly et al.	**2020**	(2)	(2)	(1)	(1)	(2)	(2)	(1)	(2)
Massahud et al	**2022**	(2)	(2)	(1)	(1)	(2)	(2)	(1)	(2)
Ning et al.	**2023**	(2)	(2)	(2)	(2)	(2)	(2)	(2)	(2)
Okura et al.	**2023**	(2)	(2)	(1)	(1)	(2)	(2)	(1)	(2)
Cid-Verdejo et al.	**2024**	(2)	(2)	(1)	(1)	(2)	(2)	(1)	(2)
Smardz et al	**2022**	(2)	(2)	(1)	(1)	(2)	(1)	(1)	(2)
Wieckiewicz et al.	**2020**	(2)	(2)	(1)	(1)	(1)	(1)	(1)	(2)

Risk-of-bias legend : (1) low (2) moderate (3) serious (4) critical (5) no information
